# Knockdown of circ_0113656 assuages oxidized low-density lipoprotein-induced vascular smooth muscle cell injury through the miR-188-3p/IGF2 pathway

**DOI:** 10.1515/med-2023-0687

**Published:** 2023-07-04

**Authors:** Ming Yang, Jun Luo, Shuhua Zhang, Qing Huang, Qianqiang Cao

**Affiliations:** Department of Vasculocardiology, People’s Hospital of Jiangxi Provincial, Nanchang, China; Department of Vasculocardioloy, People’s Hospital of Ganzhou City, Ganzhou, China; Department of Vasculocardiology, People’s Hospital of Jiangxi Provincial, No. 266, Fenhe North Road, Nanchang, China

**Keywords:** AS, circ_0113656, miR-188-3p, IGF2

## Abstract

Circular RNA (circRNA) is involved in the pathogenesis of atherosclerosis (AS). The present work analyzed the RNA expression of circ_0113656, microRNA-188-3p (miR-188-3p), and insulin-like growth factor 2 (IGF2) by quantitative real-time polymerase chain reaction. The protein expression of proliferating cell nuclear antigen (PCNA), matrix metalloprotein 2 (MMP2), and IGF2 was detected by Western blotting. Cell viability, proliferation, invasion, and migration were analyzed using the cell counting kit-8, 5-ethynyl-2′-deoxyuridine, transwell invasion, and wound-healing assays, respectively. The interactions among circ_0113656, miR-188-3p, and IGF2 were identified by dual-luciferase reporter assay and RNA immunoprecipitation assay. The results showed that circ_0113656 and IGF2 expression were significantly upregulated, while miR-188-3p was downregulated in the blood of AS patients and oxidized low-density lipoprotein (ox-LDL)-treated HVSMCs in comparison with controls. The ox-LDL treatment induced HVSMC proliferation, migration, and invasion accompanied by increases in PCNA and MMP2 expression; however, these effects were attenuated after circ_0113656 knockdown. Circ_0113656 acted as a miR-188-3p sponge and it regulated ox-LDL-induced HVSMC disorders by binding to miR-188-3p. Besides, the regulation of miR-188-3p in ox-LDL-induced HVSMC injury involved IGF2. Further, the depletion of circ_0113656 inhibited IGF2 expression by interacting with miR-188-3p. Thus, the circ_0113656/miR-188-3p/IGF2 axis may mediate ox-LDL-induced HVSMC disorders in AS, providing a new therapeutic strategy for AS.

## 1 Introduction

Cardiovascular diseases affect more than 80% of people over 85 years old and are the leading cause of death. The cause of the disease is atherosclerosis (AS), which is a lipid-driven progressive disorder [1]. AS can damage the integrity of the arterial surface and finally lead to the formation of a thrombus [2]. As reported, the pathogenesis of AS involves the accumulation of vascular smooth muscle cells (VSMCs) and lipids [3]. Oxidized low-density lipoprotein (ox-LDL) that can promote lipid deposition in the arterial wall has been considered a cardinal risk for AS progression [4]. Thus, exploring the underlying mechanism of ox-LDL-induced human vascular smooth muscle cell (HVSMC) injury may be helpful to develop new strategies for AS therapy.

Circular RNA (circRNA) is a single-stranded molecular produced by back-splicing, does not contain a 5′-cap or 3′-polyadenylation tail, and is resistant to exonuclease-mediated degradation [5]. The circular transcript modulates gene expression mainly by sponging microRNA (miRNA), interfering with pre-mRNA processing and sequestering proteins [6]. Most of the circRNAs in mammals are lowly expressed but they indeed bind to miRNAs through the complementary sites between them [7]. Multiple studies report the close association between circRNA and AS progression. For instance, circ_0002984 increases ox-LDL-induced HVSMC proliferation and inflammation by interacting with miR-326-3p [8]. Circ_0010283 combines with miR-370-3p to regulate HVSMC migration in AS [9]. Circ_0113656 is formed from exons 3 to 5 of the 24-dehydrocholesterol reductase (DHCR24) gene, which may be helpful to open up new therapeutic options for AS owing to its regulation in the cholesterol biosynthesis intermediate desmosterol [10,11]; however, the mechanism of circ_0113656 in AS occurrence is poorly known.

Representing a class of noncoding transcripts, miRNAs have ∼22 nucleotides and fine-tune cellular homeostasis by combining with their targets at the post-transcriptional level [12]. Considerable studies have disclosed the core position of these small transcripts in the pathogenesis of AS [13]. Recently, investigators explained that miR-188-3p mediated autophagy and myocardial infarction through association with long noncoding RNA (lncAPF) [14]. Lin *et al*. reported that oxygen-glucose deprivation/reoxygenation-caused HT22 cell disorders involved miR-188-3p [15]. The above evidence suggests the importance of miR-188-3p in the development of cardiovascular and cerebrovascular diseases.

We found that circ_0113656 is potentially bound to miR-188-3p using bioinformatics tools. Given that miR-188-3p contains the binding sites of insulin-like growth factor 2 (IGF2), which can regulate AS progression [16], we hypothesize that the circ_0113656/miR-188-3p/IGF2 pathway is responsible for the occurrence of AS. Thus, we analyzed the function of circ_0113656 in ox-LDL-induced HVSMC injury and determined whether the regulation of circ_0113656 in ox-LDL-induced HVSMC damage involved the circ_0113656/miR-188-3p/IGF2 pathway.

## 2 Materials and methods

### 2.1 Study subjects

Blood samples were collected from AS patients (*N* = 17) and healthy volunteers (*N* = 13) at the People’s Hospital of Jiangxi Provincial. Plaque sites and ranges were evaluated using a Madison ultrasound system. Besides, two neurologists identified the diseases. AS patients with other clinical diseases were excluded. Blood samples were centrifuged at 1,000*g* for 10 min and then stored in a refrigerator.


**Ethical approval:** The research related to human use has complied with all the relevant national regulations and institutional policies, in accordance with the tenets of the Helsinki Declaration, and has been approved by the Ethics Committee of People’s Hospital of Jiangxi Provincial. All participants signed the written informed consent.

### 2.2 Cell culture and ox-LDL stimulation

HVSMCs were purchased from Tongpai Biotech (Shanghai, China) and maintained in Ham’s F12K medium (Sunncell, Wuhan, China) plus 10% fetal bovine serum (Sunncell) and 1% penicillin/streptomycin (Sunncell) at 37°C in an incubator buffered with 5% CO_2_. To disclose the molecular mechanism behind circ_0113656 regulating HVSMC injury, HVSMCs were stimulated using ox-LDL (Yeasen Biotech, Shanghai, China; 0, 25, 50, and 100 µg/mL) for 24 h as shown previously [17].

### 2.3 Cell transfection

Small interfering RNA of circ_0113656 (si-circ_0113656, 5′-GGCAGAGCCCAGCAAGATTGT-3′), miR-188-3p mimics (miR-188-3p, 5′-CUCCCACAUGCAGGGUUUGCA-3′), miR-188-3p inhibitors (anti-miR-188-3p, 5′-UGCAAACCCUGCAUGUGGGAG-3′) and the corresponding negative controls (si-NC, miR-NC, and anti-miR-NC) were synthesized by Ribobio Co., Ltd. (Guangzhou, China). The full-length coding sequence of IGF2 was amplified and inserted into the pcDNA 3.1(+) vector to generate the overexpression plasmid of IGF2. In strict accordance with the guidebook of Lipofectamine 3000, transfection was performed on HVSMCs maintained in 12-well plates in an atmosphere containing 5% CO_2_. Transfection efficiency was evaluated by quantitative real-time polymerase chain reaction (qRT-PCR).

### 2.4 Cell viability

HVSMCs transfected with plasmids and oligonucleotides or treated with ox-LDL were maintained in 96-well plates for 48 h. Then, the cell counting kit-8 (CCK-8) reagent (Dojindo, Shanghai, China) was used to incubate the cells according to the manufacturer’s direction. Finally, these samples were analyzed using an enzyme immunoassay analyzer (Azure Ao; Cycloud Biotech, Beijing, China).

### 2.5 Cell proliferation analysis

The assay was performed to determine the proliferative ability of HVSMCs with various treatments. In brief, HVSMCs were treated and allowed to grow in 12-well plates for 48 h. The cells were subcultured in 96-well plates supplemented with EdU-labeled Ham’s F12K (Sunncell) for 2 h and then stained using 4′,6-diamidino-2-phenylindole (Sbjbio® life science, Nanjing, China). At last, the stained cells from five random fields were analyzed using a confocal microscope (Olympus, Tokyo, Japan).

### 2.6 Transwell invasion assay

Transwell analysis involving the use of transwell compartments with Matrigel (Corning, Beijing, China) was performed to evaluate the invasive capacity of HVSMCs. In brief, cells with various treatments were allowed to culture in the upper chambers, which were pre-added with serum-free Ham’s F12K medium (Sunncell), whereas the lower chambers were supplemented with Ham’s F12K medium plus 15% serum. After 24 h incubation, the cells invaded into the lower chambers and were dyed using crystal violet (Yaji Biotech, Shanghai, China) and then counted under an inverted microscope (100× magnification; Olympus).

### 2.7 Wound-healing assay

HVSMCs at 80–90% confluence were treated with plasmids, oligonucleotide, and ox-LDL alone or jointly. After 14 days of culture, sterile 10 μL tips were applied to scratch the cells, and then the cells went through 24 h culture. The images of wounds were captured at 0 and 24 h using an inverted microscope (Olympus).

### 2.8 Western blotting analysis

Total proteins from the blood samples of AS patients and HVSMCs were isolated using RIPA lysis buffer containing a protease inhibitor (Sbjbio^®^ life science). The concentrations of extracted proteins were determined with a BCA protein assay kit (Sbjbio^®^ life science). Then, polyacrylamide gels were used to separate protein with a SureLock Midi-Cell Electrophoresis System. After blocking the aspecific signals using skimmed milk (Yili, Beijing, China), polyvinylidene fluoride membranes with protein bands were incubated with the primary antibodies against proliferating cell nuclear antigen (PCNA) (#13-3900; 1:5000; Thermo Fisher, Waltham, MA, USA), matrix metalloprotein 2 (MMP2) (#436000; 1:250; Thermo Fisher), IGF2 (#MA5-17096; 1:1,000; Thermo Fisher), and glyceraldehyde 3-phosphate dehydrogenase (GAPDH) (#MA1-16757; 1:2,000; Thermo Fisher). After being exposed to eyoECL Plus (Beyotime, Shanghai, China), the visualized blots were analyzed using Image J software.

### 2.9 qRT-PCR and RNA treatment

TRIzol™ reagent (#15596026; Thermo Fisher) was used to prepare cell lysates as per the guidebook. A NanoDrop spectrophotometer was applied to detect RNA concentration. cDNA synthesis reagents (Vazyme, Jiangsu, China) and miRNA first strand synthesis kit (Vazyme) were used for reverse transcription. Then, diluted cDNA was subjected to qRT-PCR analysis on a qRT-PCR system (Bio-Rad, Hercules, CA, USA) with SYBR qPCR Master Mix (Vazyme). The expression of circ_0113656, miR-188-3p, and IFG2 was normalized to GAPDH or U6 by the 2^−∆∆Ct^ method. Random primers and oligo(dT)_18_ primers were utilized to identify the circular structure of circ_0113656. Besides, 1 μg of RNA was exposed to RNase R (Xiyuan Biotech, Shanghai, China) at 37°C for 20 min to analyze the circRNA structure. Primers used for amplification are listed in Table 1.

**Table 1:** Primer sequences used for qRT-PCR

Name		Sequences (5′–3′)
circ_0113656	Forward	GAGCCCAGCAAGATTGTCCGT
	Reverse	TCATCAAGCTCAGGCAACACG
miR-188-3p	Forward	ATTATTGGCTCCCACATGCAG
	Reverse	ATCCAGTGCAGGGTCCGAGG
IGF2	Forward	TCGCCGAACCAAAGTGGATTA
	Reverse	GGGAGAGACAGAGTGAACGTG
GAPDH	Forward	CAAATTCCATGGCACCGTCA
	Reverse	GACTCCACGACGTACTCAGC
U6	Forward	CTTCGGCAGCACATATACT
	Reverse	AAAATATGGAACGCTTCACG

### 2.10 RNA immunoprecipitation (RIP) assay

The assay was performed based on the guidebook of a Magna RIP kit (Sigma, St. Louis, MO, USA). In brief, 1 × 10^5^ HVSMCs cultured in 12-well plates were collected and lysed using RIP lysis buffer (Sbjbio^®^ life science). Then, the lysates were incubated with anti-Ago2-conjugated magnetic beads for 24 h with an IgG antibody as a negative control. Finally, the expression of circ_0113656, miR-188-3p, and IGF2 in the complexes enriched with magnetic beads was quantified by qRT-PCR.

### 2.11 Dual-luciferase reporter assay

The binding sites of miR-188-3p for circ_0113656 and IFG2 were predicted using online databases including Circinteractome (https://circinteractome.nia.nih.gov/index.html), Circbank (http://www.circbank.cn/index.html), and Targetscan (http://www.targetscan.org/vert_71/). The wild-type (WT) reporter plasmids including WT-circ_0113656 and WT-IGF2 3′-untranslated region (3′-UTR) were generated by introducing the sequences of circ_0113656 and IGF2 3′-UTR with the binding sites of miR-188-3p into the pmirGLO vector. The mutant plasmids including MUT-circ_0113656 and MUT-IGF2 3′-UTR were built using site mutagenesis kits (Yeasen Biotech). Then, the reporter plasmids together with miR-188-3p mimics or mimic control were transfected into HVSMCs for 48 h. At last, the dual-Lucy assay kit (Solarbio, Beijing, China) was utilized to analyze the binding intensity.

### 2.12 Statistical analysis

All data from three independent duplicate tests were analyzed by GraphPad Prism and presented as mean ± standard deviations (SD). The significant differences were compared with Mann–Whitney *U* test, Student’s *t*-test, or analysis of variance. Spearman correlation analysis was used to analyze the interactions of miR-188-3p expression with circ_0113656 and IGF2 expression. *P* < 0.05 indicated a statistical significance.

## 3 Results

### 3.1 Ox-LDL treatment induces HVSMC injury

**Figure 1:**
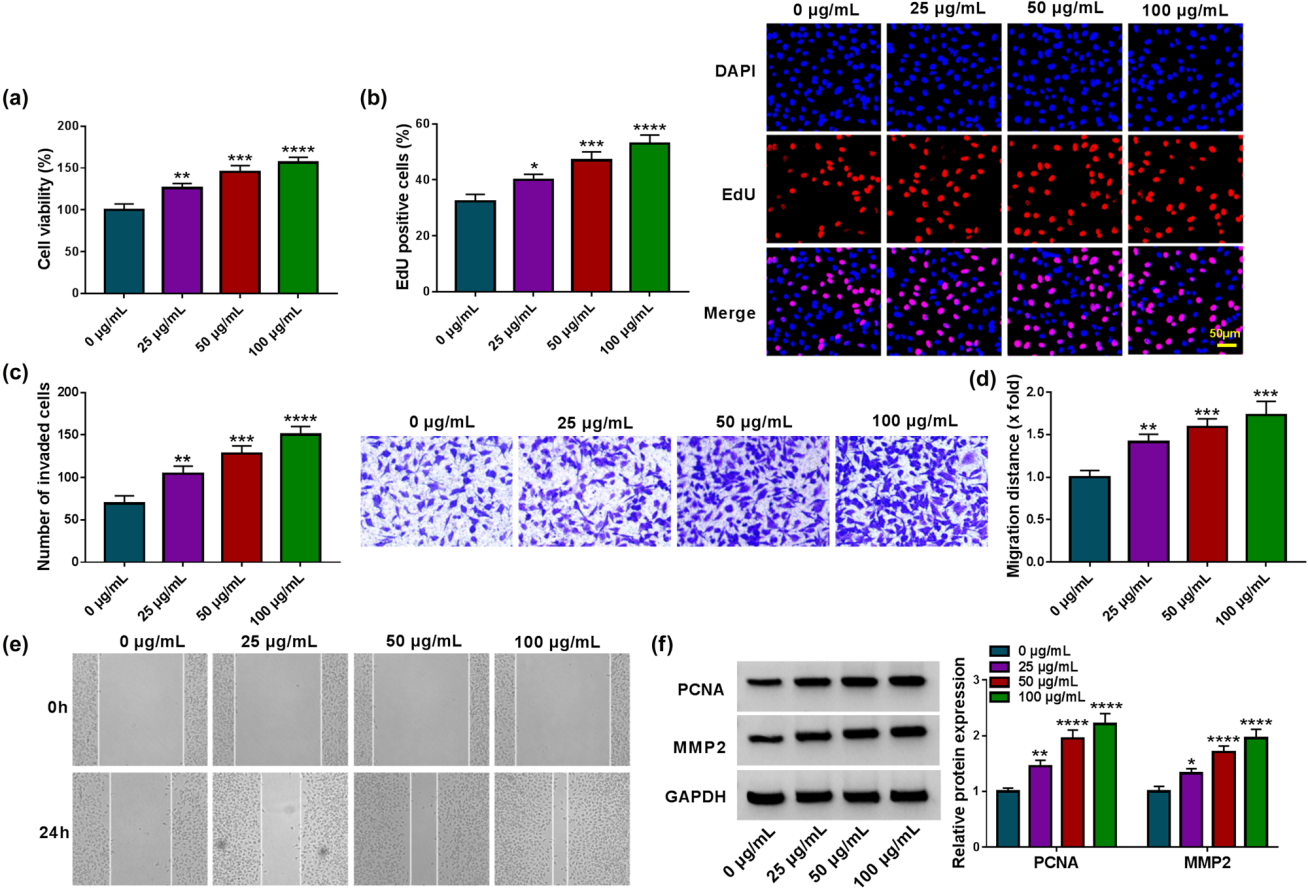
Ox-LDL induces HVSMC injury. HVSMCs were treated with various concentrations of ox-LDL (0, 25, 50, and 100 µg/mL), and cell viability was analyzed by CCK-8 (a), cell proliferation by EdU assay (b), cell invasion by transwell assay (c), cell migration by wound-healing assay (d and e), and the protein expression of PCNA and MMP2 by Western blotting analysis (f). *P < 0.05, **P < 0.01, ***P < 0.001, and ****P < 0.0001.

HVSMCs cells were treated with ox-LDL at various concentrations (0, 25, 50, and 100 µg/mL) for 24 h to mimic AS-like injury *in vivo* and explore the consequential effects on cell proliferation, invasion, and migration. As shown in Figure 1a and b, ox-LDL treatment dose-dependently promoted HVSMC viability and proliferation. Consistently, the invasion and migration of HVSMCs were increased after ox-LDL treatment in a dose-dependent manner (Figure 1c–e). As expected, the expression of the two proteins was dose-dependently upregulated by ox-LDL treatment (Figure 1f). These results demonstrate that ox-LDL treatment indeed induces HVSMC injury. Based on the above results, HVSMCs were treated with 50 µg/mL ox-LDL for 24 h in the following study.

### 3.2 Circ_0113656 knockdown assuages ox-LDL-induced HVSMC disorders

**Figure 2:**
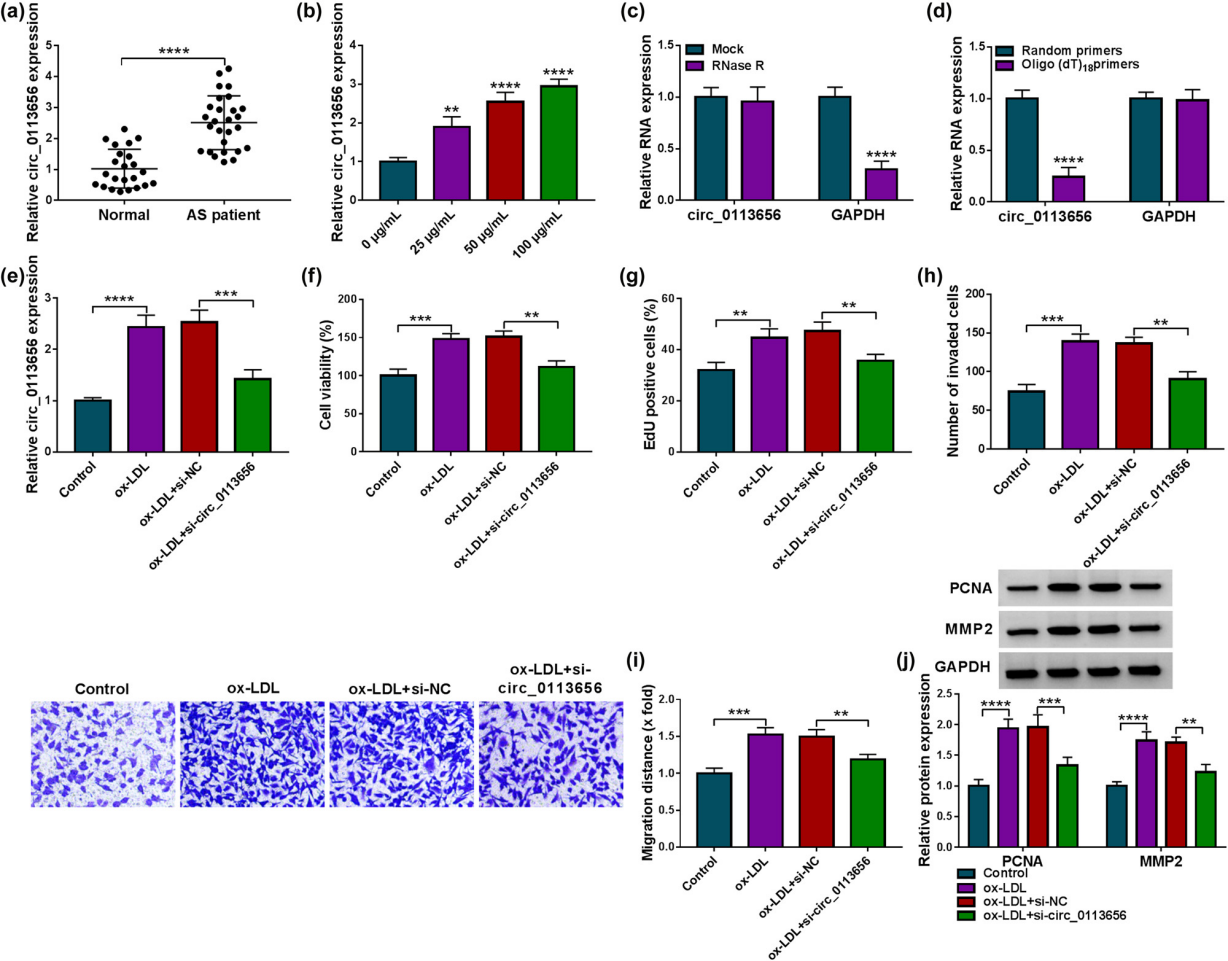
The effects of circ_0113656 on ox-LDL-induced HVSMC injury. (a) Circ_0113656 expression was detected by qRT-PCR in the blood samples of AS patients and healthy volunteers (*N* = 17 and *N* = 13, respectively). (b) Circ_0113656 expression was detected by qRT-PCR in HVSMCs treated with ox-LDL (0, 25, 50, and 100 µg/mL). (c and d) The circular structure of circ_0113656 was identified by using RNase R, random primers, and oligo(dT)_18_ primers. HVSMCs were divided into the ox-LDL group, ox-LDL + si-NC group, and ox-LDL + si-circ_0113656 group, with untreated HVSMCs as controls, and circ_0113656 expression was analyzed by qRT-PCR (e), cell viability by CCK-8 (f), cell proliferation by EdU assay (g), cell invasion by transwell assay (h), cell migration by wound-healing assay (i), and the protein expression of PCNA and MMP2 by Western blotting analysis (j). **P < 0.01, ***P < 0.001, and ****P < 0.0001.

Then, we detected circ_0113656 expression in the blood samples of AS patients. As shown in Figure 2a, circ_0113656 expression was significantly increased in the blood of AS patients in comparison with healthy controls. Moreover, circ_0113656 expression was increased in ox-LDL-stimulated HVSMCs in a concentration-dependent manner (Figure 2b). Subsequently, our results identified the circular structure of circ_0113656 using RNase R, random primers, and oligo(dT)_18_ primers. For instance, RNase R treatment greatly reduced GAPDH expression but it had no significant effect on circ_0113656 expression (Figure 2c). Meanwhile, PCR production of circ_0113656 amplified using random primers was more than that amplified using oligo(dT)_18_ primers (Figure 2d). Based on the above results, we silenced circ_0113656 in ox-LDL-treated HVSMCs to analyze the consequent effects on cell proliferation, invasion, and migration. The results of qRT-PCR showed that ox-LDL treatment increased circ_0113656 expression, which was attenuated after circ_0113656 depletion (Figure 2e). Comparatively, ox-LDL-induced promotion in cell viability, proliferation, invasion, and migration were relieved after circ_0113656 silencing (Figure 2f–i). Besides, the increased expression of PCNA and MMP2 by ox-LDL was remitted when circ_0113656 expression was downregulated (Figure 2j).

### 3.3 Circ_0113656 acts as a miR-188-3p sponge

**Figure 3:**
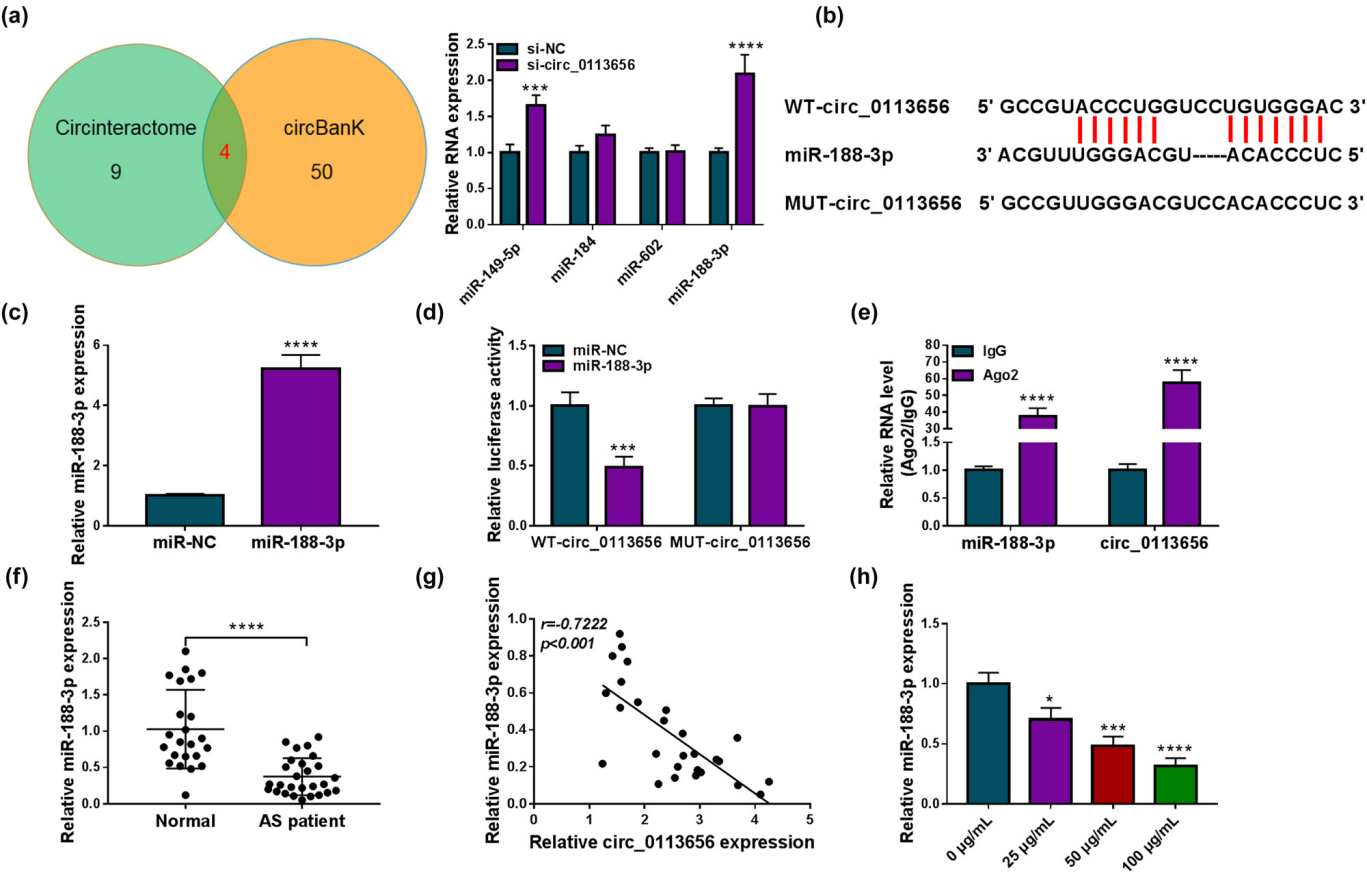
MiR-188-3p targets circ_0113656 in HVSMCs. (a) The miRNAs containing the complementary sites of circ_0113656 were predicted by Circinteractome and circBank online databases. (b) Schematic illustration showing the binding sites of circ_0113656 for miR-188-3p. (c) The efficiency of miR-188-3p mimics in increasing miR-188-3p expression was detected by qRT-PCR in HVSMCs. (d and e) Dual-luciferase reporter assay and RIP assay were carried out to determine the association between miR-188-3p and circ_0113656. (f) MiR-188-3p expression was detected by qRT-PCR in the blood samples of AS patients and healthy volunteers (*N* = 17 and *N* = 13, respectively). (g) Spearman correlation analysis was used to determine the linear correlation between miR-188-3p and circ_0113656 expression in the blood samples of AS patients. (h) MiR-188-3p expression was checked by qRT-PCR in HVSMCs treated with ox-LDL (0, 25, 50, and 100 µg/mL). *P < 0.05, ***P < 0.001, and ****P < 0.0001.

Circular RNA interactome (Circinteractome) and circBank online databases were used to predict the target miRNAs of circ_0113656. As presented in Figure 3a, we found four miRNAs that contained the complementary sites of circ_0113656, including miR-149-5p, miR-184, miR-602, and miR-188-3p, by overlapping the prediction results of the two online databases. Given higher miR-188-3p expression in HVSMCs transfected with si-circ_0113656 (Figure 3a), the miRNA was employed as a follow-up subject. The binding sites of circ_0113656 for miR-188-3p are shown in Figure 3b. Subsequently, we identified the regulatory relationship of circ_0113656 for miR-188-3p using dual-luciferase reporter assay and RIP assay. The success of miR-188-3p overexpression is presented in Figure 3c. As shown in Figure 3d, miR-188-3p introduction significantly inhibited the luciferase activity of wt-circ_0113656 but not that of mut-circ_0113656. Also, miR-188-3p and circ_0113656 could be greatly enriched in the Ago2 antibody group in comparison with the IgG antibody group (Figure 3e). We observed that miR-188-3p expression was significantly downregulated and was negatively correlated with circ_0113656 expression in the blood samples of AS patients (Figure 3f and g). Further, ox-LDL treatment reduced miR-188-3p expression in HVSMCs (Figure 3h) in a concentration-dependent manner.

### 3.4 Circ_0113656 regulates ox-LDL-induced HVSMC disorders by binding to miR-188-3p

**Figure 4:**
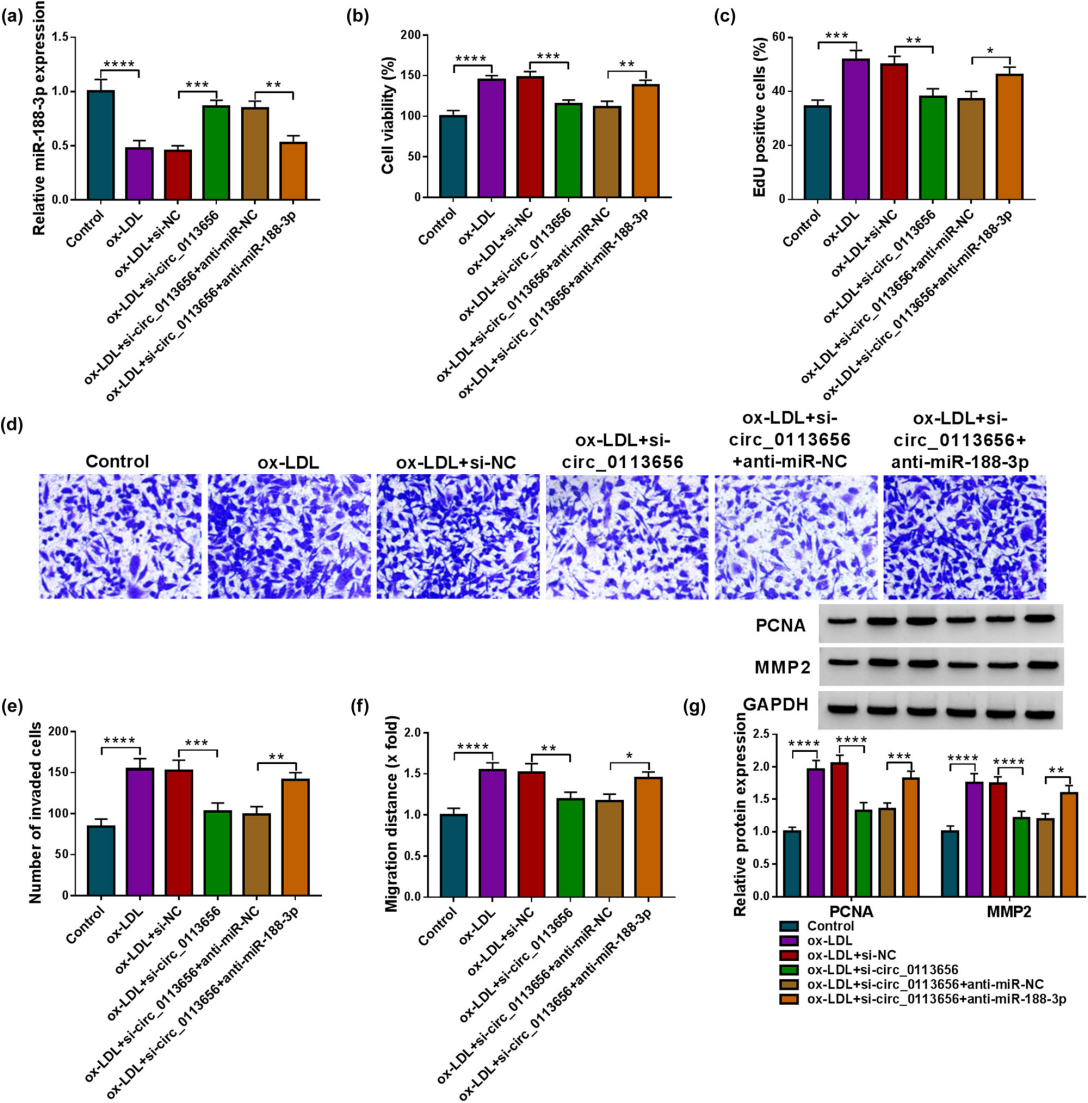
The effects of circ_0113656 knockdown and miR-188-3p depletion on ox-LDL-induced HVSMC injury. HVSMCs were divided into the ox-LDL group, ox-LDL + si-NC group, ox-LDL + si-circ_0113656 group, ox-LDL + si-circ_0113656 + anti-miR-NC group, and ox-LDL + si-circ_0113656 + anti-miR-188-3p group, with untreated HVSMCs as controls. MiR-188-3p expression was analyzed by qRT-PCR (a), cell viability by CCK-8 (b), cell proliferation by EdU assay (c), cell invasion by transwell assay (d and e), cell migration by wound-healing assay (f), and the protein expression of PCNA and MMP2 by Western blotting analysis (g). *P < 0.05, **P < 0.01, ***P < 0.001, and ****P < 0.0001.

We silenced circ_0113656 and miR-188-3p to analyze the consequential effects in ox-LDL-induced HVSMCs. As presented in Figure 4a, circ_0113656 knockdown-induced upregulation of miR-188-3p was attenuated after miR-188-3p depletion in ox-LDL-treated HVSMCs. Subsequently, we observed that circ_0113656 depletion-induced inhibition in cell viability, proliferation, invasion, and migration were remitted when miR-188-3p expression was downregulated in ox-LDL-treated HVSMCs (Figure 4b–f). Circ_0113656 knockdown significantly reduced PCNA and MMP2 expression in ox-LDL-treated HVSMCs, whereas these effects were rescued after transfection with miR-188-3p inhibitors (Figure 4g).

### 3.5 MiR-188-3p binds to IGF2 in HVSMCs

**Figure 5:**
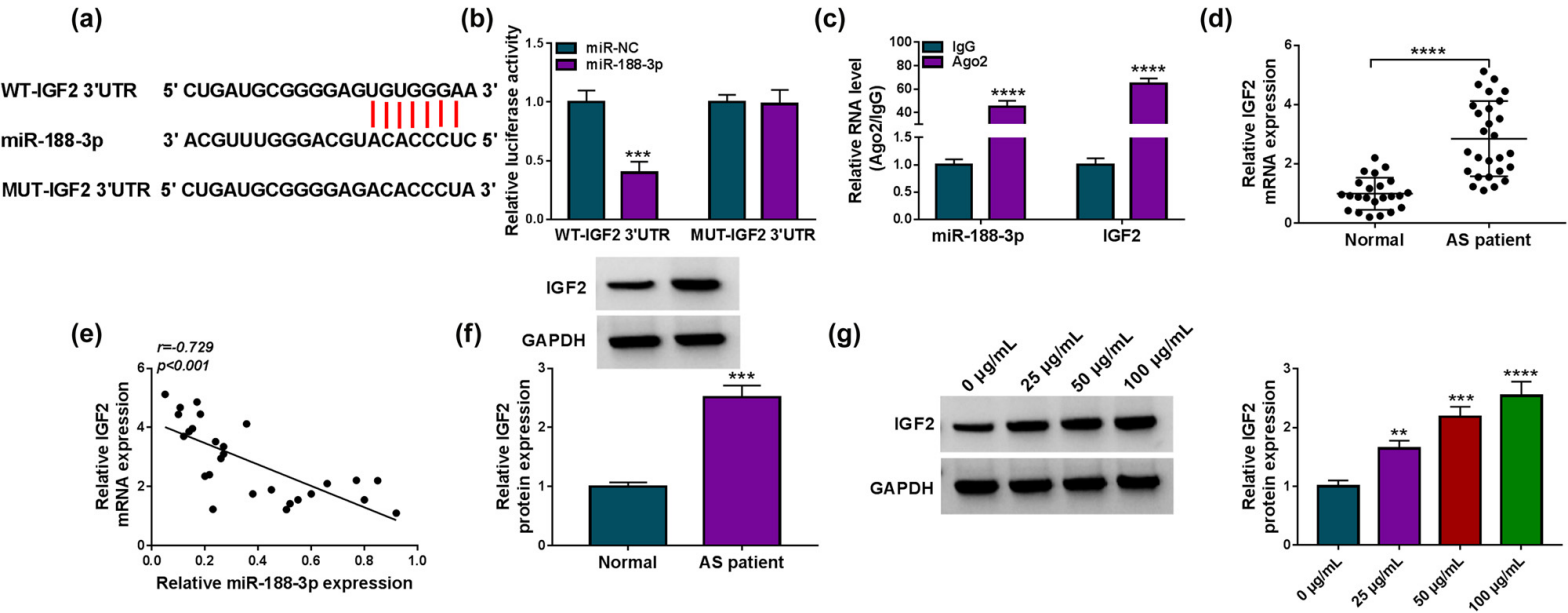
IGF2 is identified as a target of miR-188-3p. (a) Schematic illustration showing the complementary sites of miR-188-3p with IGF2. (b and c) Dual-luciferase reporter assay and RIP assay were performed to analyze the association between miR-188-3p and IGF2. (d and f) The mRNA and protein expression of IGF2 was detected by qRT-PCR and Western blotting analysis, respectively, in the blood samples from AS patients and healthy volunteers. (e) Spearman correlation analysis was used to determine the linear correlation between miR-188-3p and IGF2 expression in the blood samples of AS patients. (g) IGF2 protein expression was checked by Western blotting analysis in HVSMCs treated with ox-LDL (0, 25, 50, and 100 µg/mL). **P < 0.01, ***P < 0.001, and ****P < 0.0001.

As predicted by Targetscan online database, IGF2 contained the binding sites of miR-188-3p (Figure 5a). We then performed dual-luciferase reporter assay and RIP assay to determine whether miR-188-3p targeted IGF2. As expected, the luciferase activity of WT-IGF2 3′-UTR was significantly reduced after transfection with miR-188-3p mimics, whereas that of MUT-IGF2 3′-UTR had no response to miR-188-3p introduction (Figure 5b). Meanwhile, miR-188-3p and IGF2 expression in the co-precipitated RNAs induced by the Ago2 antibody were higher than their expression in the co-precipitated RNAs induced by the IgG antibody (Figure 5c). These data suggest that miR-188-3p targets IGF2. Consistently, we found that IGF2 expression was greatly increased and negatively correlated with miR-188-3p expression in the blood samples of AS patients (Figure 5d–f). Further, ox-LDL treatment promoted IGF2 production in a concentration-dependent manner (Figure 5g).

### 3.6 IGF2 overexpression remits miR-188-3p-mediated effects in ox-LDL-treated HVSMCs

**Figure 6:**
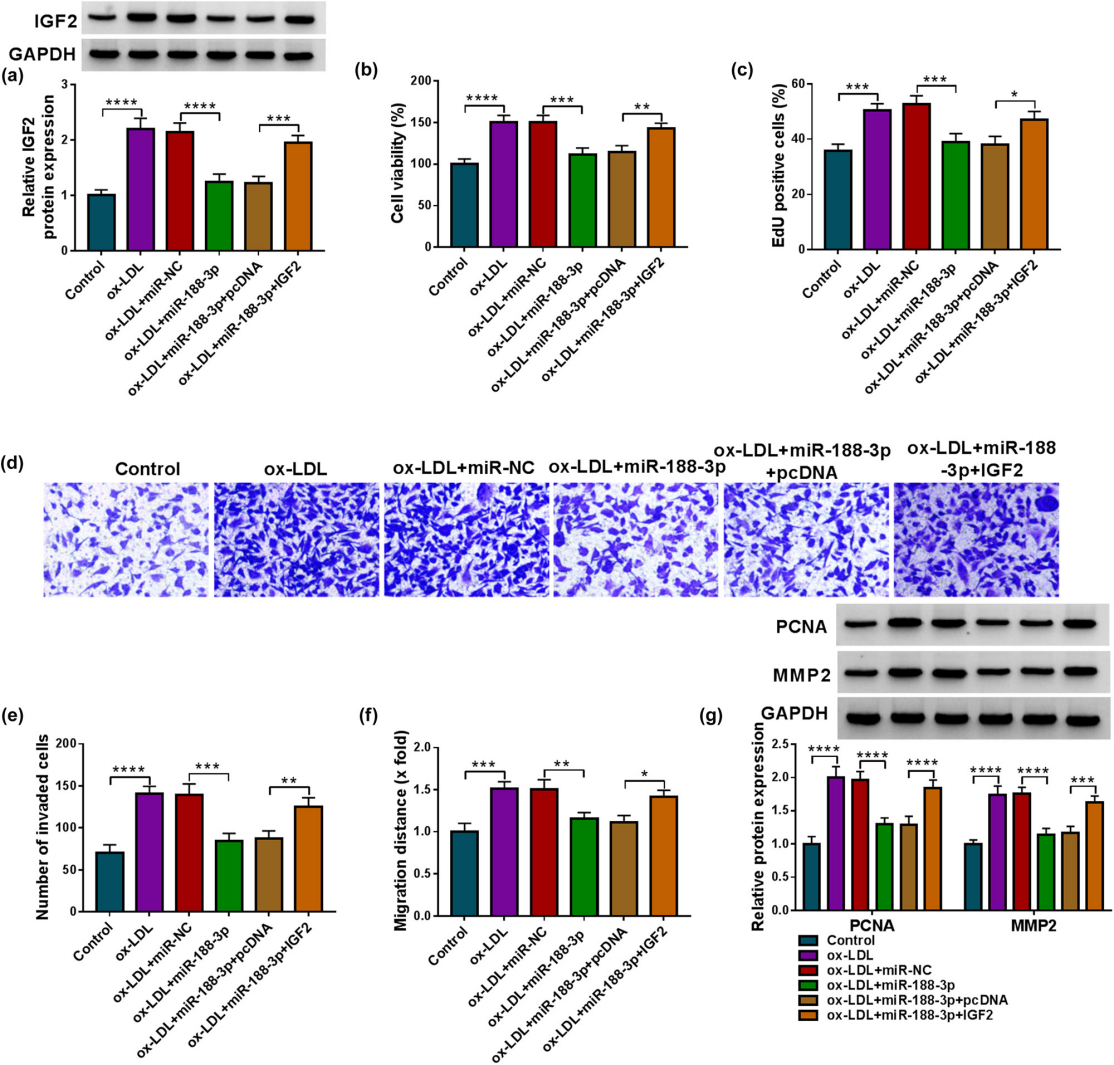
The effects of miR-188-3p and IGF2 on ox-LDL-induced HVSMC disorders. HVSMCs were divided into the ox-LDL group, ox-LDL + miR-NC group, ox-LDL + miR-188-3p group, ox-LDL + miR-188-3p + pcDNA group, and ox-LDL + miR-188-3p + IGF2 group, with mock HVSMCs as controls. IGF2 expression was analyzed by Western blotting analysis (a), cell viability by CCK-8 (b), cell proliferation by EdU assay (c), cell invasion by transwell assay (d and e), cell migration by wound-healing assay (f), and the protein expression of PCNA and MMP2 by Western blotting analysis (g). *P < 0.05, **P < 0.01, ***P < 0.001, and ****P < 0.0001.

**Figure A1:**
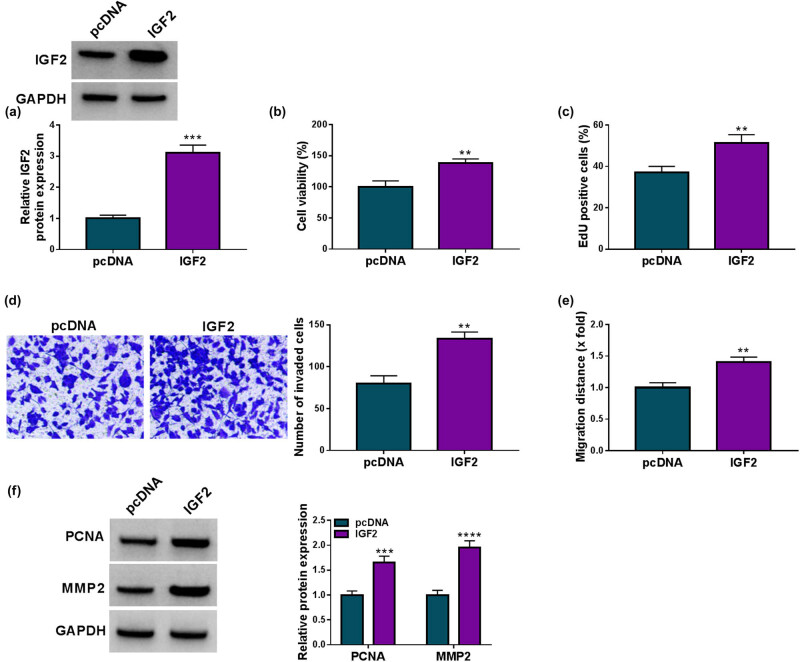
The effects of IGF2 overexpression on HVSMC phenotypes. HVSMCs were transfected with pcDNA or the overexpression plasmid of IGF2, and IGF2 expression was analyzed by Western blotting analysis (a), cell viability by CCK-8 (b), cell proliferation by EdU assay (c), cell invasion by transwell assay (d), cell migration by wound-healing assay (e), and the protein expression of PCNA and MMP2 by Western blotting analysis (f). **P < 0.01, and ***P < 0.001.

Given the association between miR-188-3p and IGF2, we further analyzed whether IGF2 participated in miR-188-3p-induced effects in ox-LDL-treated HVSMCs. The study first showed that miR-188-3p mimics reduced IGF2 expression in ox-LDL-treated HVSMCs, whereas the effect was attenuated after IGF2 overexpression (Figure 6a). Subsequently, miR-188-3p introduction inhibited cell viability, proliferation, invasion, and migration; however, these effects were remitted when IGF2 was overexpressed (Figure 6b–f). In addition, miR-188-3p-induced inhibition in PCNA and MMP2 expression was restored by the enforced IGF2 expression (Figure 6g). Moreover, we analyzed the effect of IGF2 overexpression on the change of HVSMC phenotypes. The efficiency of IGF2 overexpression is shown in Figure A1a. Then, the data showed that IGF2 overexpression promoted HVSMC viability, proliferation, invasion, migration, and the protein expression of PCNA and MMP2 (Figure A1b–f).

### 3.7 Circ_0113656 depletion reduces IGF2 expression through miR-188-3p in ox-LDL-treated HVSMCs

**Figure 7:**
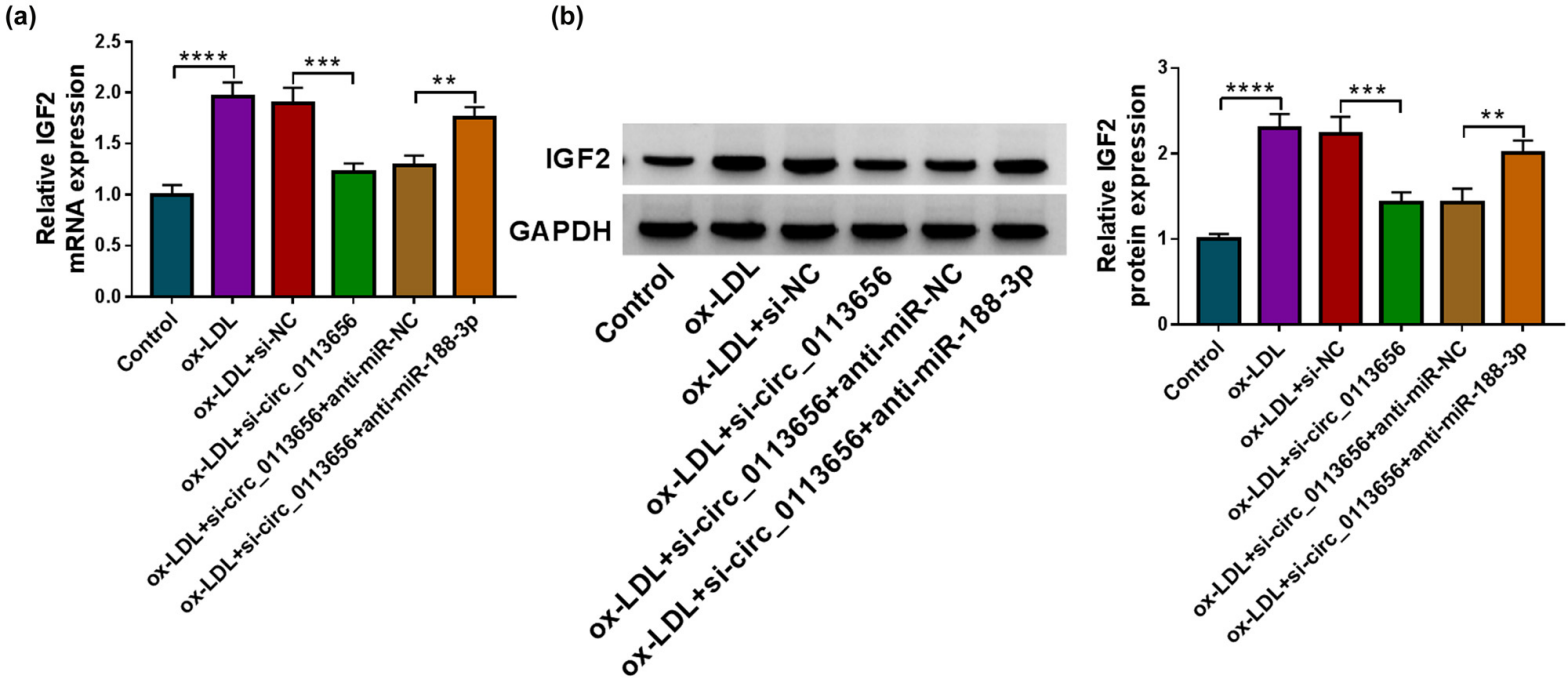
The effects of circ_0113656 knockdown and miR-188-3p depletion on IGF2 expression in ox-LDL-treated HVSMCs. HVSMCs were divided into the ox-LDL group, ox-LDL + si-NC group, ox-LDL + si-circ_0113656 group, ox-LDL + si-circ_0113656 + anti-miR-NC group, and ox-LDL + si-circ_0113656 + anti-miR-188-3p group, with untreated HVSMCs as controls. IGF2 mRNA expression was detected by qRT-PCR (a) and IGF2 protein expression by Western blotting analysis (b). **P < 0.01, ***P < 0.001, and ****P < 0.0001.

Based on the above findings, we continued to explore whether IGF2 was the downstream gene of the circ_0113656/miR-188-3p axis in ox-LDL-treated HVSMCs. We silenced circ_0113656 and miR-188-3p in the cells and then detected IGF2 expression using qRT-PCR and Western blotting analysis. As presented in Figure 7a and b, circ_0113656 knockdown significantly reduced the mRNA and protein expression of IGF2 with ox-LDL treatment; however, these effects were remitted when miR-188–3p expression was downregulated. These results demonstrate that circ_0113656 regulates IGF2 expression through miR-188-3p.

## 4 Discussion

In the present study, we found that circ_0113656 expression was significantly upregulated in the blood of AS patients and ox-LDL-treated HVSMCs in comparison with controls. Ox-LDL exposure induced HVSMC proliferation, migration, and invasion but these effects were relieved when circ_0113656 expression was downregulated. Circ_0113656 modulated ox-LDL-induced HVSMC disorders by binding to miR-188-3p. Moreover, the regulation of miR-188-3p in ox-LDL-induced HVSMC injury involved IGF2. Further, the depletion of circ_0113656 inhibited IGF2 expression by interacting with miR-188-3p.

AS is a life-long disorder of large- and medium-sized arteries and can cause cardiovascular diseases like ischemic heart disease and stroke [18]. Ox-LDL is a circulating biomarker that can stimulate the production of endothelin-1 and matrix-degrading enzymes, induce the apoptosis of VSMCs, and promote the synthesis of collagen by VSMCs and fibroblasts, thus regulating the production of proinflammatory genes and the transformation of fatty plaques into AS [19]. The latest research progress indicates that circRNAs regulate the biological behaviors of HVSMCs and macrophages during AS [20]. In particular, considerable data have explained the involvement of circRNAs such as circ_0044073 [21], circRNA homeodomain interacting protein kinase 3 (circHIPK3) [22], and circ_0006896 [23] in AS. Circ_0113656 is also named circDHCR24, and its knockdown inhibits the proliferation, migration, and phenotypic switch of platelet-derived growth factor-BB-treated HVSMCs [24]. Herein, we reported that circ_0113656 was overexpressed in AS patients and ox-LDL-induced HVSMCs and that circ_0113656 depletion assuaged ox-LDL-induced HVSMC proliferation. PCNA is an important factor that forms a homotrimeric ring embracing DNA and regulates DNA replication and repair [25]. MMP2 is a zinc-dependent endopeptidase that can degrade IV collagen and is associated with metastasis formation [26]. In this work, we found that circ_0113656 silencing remitted ox-LDL-induced HVSMC invasion and migration as well as the production of PCNA and MMP2. The above data indicate that circ_0113656 acts as a pathogenic gene in AS progression.

MiRNA comprises 18–25 nucleotides and has received much attention for its roles in tempering gene expression. More and more studies are carried out to emphasize the importance of miRNA in AS progression [13]. As reported, miRNAs can affect the formation of foam cells, which are associated with plaque formation [27]. Besides, several investigators have demonstrated that miRNAs mediate ABC transporter expression, intra-arterial macrophage migration, and VSMC differentiation [28,29]. MiR-188-3p plays important roles in cancers [30], polycystic ovary syndrome [31], and Parkinson’s disease [32]. Recently, researchers analyzed the role of miR-188-3p in AS and found that miR-188-3p reduced intravascular lipid accumulation, repressed HVSMC proliferation and migration, and induced HVSMC apoptosis [33,34]. In this work, miR-188-3p is bound to circ_0113656 and negatively regulated by circ_0113656. Besides, miR-188-3p was weakly expressed in AS patients and ox-LDL-treated HVSMCs. Based on the above evidence, we explored whether circ_0113656 regulated ox-LDL-caused cell disorders through miR-188-3p. As expected, miR-188-3p knockdown relieved circ_0113656 depletion-mediated effects in ox-LDL-treated HVSMCs, suggesting that the regulation of circ_0113656 for HVSMC injury involved miR-188-3p.

IGF2 is a protein hormone and belongs to the IGF system, serving important roles in the progression of various human diseases, including AS. As reported, IGF2 may mediate PCNA expression through the PI3K/AKT/mTOR signaling pathway [35,36]. In addition, IGF participates in remodeling by interplaying with MMPs [37]. MMPs can increase the bioavailability of IGFs for receptor activation by releasing them from association with the extracellular matrix [38]. IGF2 can stimulate VSMC proliferation and migration by binding to miR-637 [17]. IGF2 also induces VSMC proliferation and inhibits apoptosis through interaction with miR-148b [39]. In addition, Wang *et al*. reported that ox-LDL treatment increased interleukin 6 production through association with IGF2 in HP-1 macrophages [40]. Qiao *et al*. ascertained that IGF2 regulated AS-induced lipid accumulation and inflammation through association with miR-210-3p [16]. Here, we identified that IGF2 is bound to miR-188-3p. We found that IGF2 introduction remitted the inhibitory effects of miR-188-3p overexpression on HVSMC injury. Meanwhile, it was found that circ_0113656 modulated IGF2 expression through miR-188-3p.

Thus, our study showed for the first time that circ_0113656 silencing attenuated ox-LDL-induced HVSMC proliferation, migration, and invasion through the miR-188-3p/IGF2 axis. However, the regulatory role of the circ_0113656/miR-188-3p/IGF2 pathway in HVSMC disorders is only analyzed in a cell model and should be further validated using ApoE-knockout (ApoE−/−) mice. In addition, the role of the circ_0113656/miR-188–3p/IGF2 pathway in AS occurrence is analyzed using only HVSMCs and needs to be explored using other types of cells.

**Figure 8:**
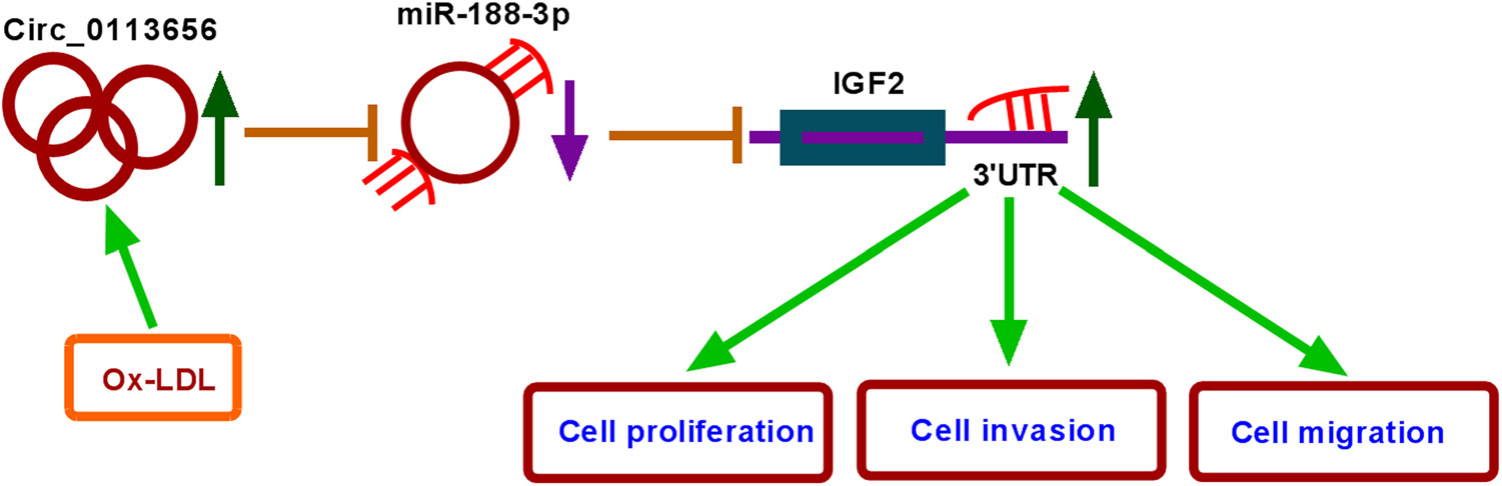
The mechanism of circ_0113656 regulating ox-LDL-induced VSMC injury.

Taken together, our findings indicate that the therapeutic potential of circ_0113656 inhibitors for AS lies in its inhibitory effects on HVSMC proliferation, migration, and invasion through the miR-188–3p/IGF2 axis (Figure 8). This work suggests that the inhibitors of circ_0113656 may be effective agents for AS therapy.

## References

[1] Libby P, Bornfeldt KE, Tall AR. Atherosclerosis: successes, surprises, and future challenges. Circ Res. 2016;118(4):531–4. 10.1161/circresaha.116.308334. PMC4762065 26892955

[2] Hansson GK, Libby P, Tabas I. Inflammation and plaque vulnerability. J Intern Med. 2015;278(5):483–93. 10.1111/joim.12406. PMC5082111 26260307

[3] Nabel EG, Braunwald E. A tale of coronary artery disease and myocardial infarction. N Engl J Med. 2012;366(1):54–63. 10.1056/NEJMra1112570. 22216842

[4] Takenaka T, Takahashi K, Kobayashi T, Oshima E, Iwasaki S, Suzuki H. Oxidized low density lipoprotein (Ox-LDL) as a marker of atherosclerosis in hemodialysis (HD) patients. Clin Nephrol. 2002;58(1):33–7. 10.5414/cnp58033. 12141404

[5] Kristensen LS, Andersen MS, Stagsted LVW, Ebbesen KK, Hansen TB, Kjems J. The biogenesis, biology and characterization of circular RNAs. Nat Rev Genet. 2019;20(11):675–91. 10.1038/s41576-019-0158-7. 31395983

[6] Chen LL. The biogenesis and emerging roles of circular RNAs. Nat Rev Mol Cell Biol. 2016;17(4):205–11. 10.1038/nrm.2015.32. 26908011

[7] Guo JU, Agarwal V, Guo H, Bartel DP. Expanded identification and characterization of mammalian circular RNAs. Genome Biol. 2014;15(7):409. 10.1186/s13059-014-0409-z. PMC4165365 25070500

[8] Li R, Jiang Q, Zheng Y. Circ_0002984 induces proliferation, migration and inflammation response of VSMCs induced by ox-LDL through miR-326-3p/VAMP3 axis in atherosclerosis. J Cell Mol Med. 2021;25(16):8028–38. 10.1111/jcmm.16734. PMC8358879 34169652

[9] Ding P, Ding Y, Tian Y, Lei X. Circular RNA circ_0010283 regulates the viability and migration of oxidized low‑density lipoprotein‑induced vascular smooth muscle cells via an miR‑370‑3p/HMGB1 axis in atherosclerosis. Int J Mol Med. 2020;46(4):1399–408. 10.3892/ijmm.2020.4703. PMC7447304 32945389

[10] Müller C, Hemmers S, Bartl N, Plodek A, Körner A, Mirakaj V, et al. New chemotype of selective and potent inhibitors of human delta 24-dehydrocholesterol reductase. Eur J Med Chem. 2017;140:305–20. 10.1016/j.ejmech.2017.08.011. 28964935

[11] Bae SH, Paik YK. Cholesterol biosynthesis from lanosterol: development of a novel assay method and characterization of rat liver microsomal lanosterol delta 24-reductase. Biochem J. 1997;326(Pt 2):609–16. 10.1042/bj3260609. PMC1218712 9291139

[12] Gebert LFR, MacRae IJ. Regulation of microRNA function in animals. Nat Rev Mol Cell Biol. 2019;20(1):21–37. 10.1038/s41580-018-0045-7. PMC6546304 30108335

[13] Lu Y, Thavarajah T, Gu W, Cai J, Xu Q. Impact of miRNA in Atherosclerosis. Arterioscler Thromb Vasc Biol. 2018;38(9):e159–70. 10.1161/atvbaha.118.310227. PMC6795547 30354259

[14] Wang K, Liu CY, Zhou LY, Wang JX, Wang M, Zhao B, et al. APF lncRNA regulates autophagy and myocardial infarction by targeting miR-188-3p. Nat Commun. 2015;6:6779. 10.1038/ncomms7779. 25858075

[15] Lin SP, Ye S, Long Y, Fan Y, Mao HF, Chen MT, et al. Circular RNA expression alterations are involved in OGD/R-induced neuron injury. Biochem Biophys Res Commun. 2016;471(1):52–6. 10.1016/j.bbrc.2016.01.183. 26845359

[16] Qiao XR, Wang L, Liu M, Tian Y, Chen T. MiR-210-3p attenuates lipid accumulation and inflammation in atherosclerosis by repressing IGF2. Biosci Biotechnol Biochem. 2020;84(2):321–9. 10.1080/09168451.2019.1685370. 31680642

[17] Yang N, Dong B, Song Y, Li Y, Kou L, Yang J, et al. Downregulation of miR-637 promotes vascular smooth muscle cell proliferation and migration via regulation of insulin-like growth factor-2. Cell Mol Biol Lett. 2020;25:30. 10.1186/s11658-020-00222-z. PMC7203897 32399056

[18] Weber C, Noels H. Atherosclerosis: current pathogenesis and therapeutic options. Nat Med. 2011;17(11):1410–22. 10.1038/nm.2538. 22064431

[19] Trpkovic A, Resanovic I, Stanimirovic J, Radak D, Mousa SA, Cenic-Milosevic D, et al. Oxidized low-density lipoprotein as a biomarker of cardiovascular diseases. Crit Rev Clin Lab Sci. 2015;52(2):70–85. 10.3109/10408363.2014.992063. 25537066

[20] Cao Q, Guo Z, Du S, Ling H, Song C. Circular RNAs in the pathogenesis of atherosclerosis. Life Sci. 2020;255:117837. 10.1016/j.lfs.2020.117837. 32450175

[21] Shen L, Hu Y, Lou J, Yin S, Wang W, Wang Y, et al. CircRNA‑0044073 is upregulated in atherosclerosis and increases the proliferation and invasion of cells by targeting miR‑107. Mol Med Rep. 2019;19(5):3923–32. 10.3892/mmr.2019.10011. 30864721

[22] Wei MY, Lv RR, Teng Z. Circular RNA circHIPK3 as a novel circRNA regulator of autophagy and endothelial cell dysfunction in atherosclerosis. Eur Rev Med Pharmacol Sci. 2020;24(24):12849–58. 10.26355/eurrev_202012_24187. 33378035

[23] Wen Y, Chun Y, Lian ZQ, Yong ZW, Lan YM, Huan L, et al. circRNA‑0006896‑miR1264‑DNMT1 axis plays an important role in carotid plaque destabilization by regulating the behavior of endothelial cells in atherosclerosis. Mol Med Rep. 2021;23(5):311. 10.3892/mmr.2021.11950. PMC7974330 33649864

[24] Peng W, Li T, Pi S, Huang L, Liu Y. Suppression of circular RNA circDHCR24 alleviates aortic smooth muscle cell proliferation and migration by targeting miR-149-5p/MMP9 axis. Biochem Biophys Res Commun. 2020;529(3):753–9. 10.1016/j.bbrc.2020.06.067. 32736703

[25] González-Magaña A, Blanco FJ. Human PCNA Structure, Function and Interactions. Biomolecules. 2020;10(4):570. 10.3390/biom10040570. PMC7225939 32276417

[26] Wang M, Wang T, Liu S, Yoshida D, Teramoto A. The expression of matrix metalloproteinase-2 and -9 in human gliomas of different pathological grades. Brain Tumor Pathol. 2003;20(2):65–72. 10.1007/bf02483449. 14756443

[27] Eken SM, Jin H, Chernogubova E, Li Y, Simon N, Sun C, et al. MicroRNA-210 Enhances Fibrous Cap Stability in Advanced Atherosclerotic Lesions. Circ Res. 2017;120(4):633–44. 10.1161/circresaha.116.309318. 27895035

[28] Karunakaran D, Rayner KJ. Macrophage miRNAs in atherosclerosis. Biochim Biophys Acta. 2016;1861(12 Pt B):2087–93. 10.1016/j.bbalip.2016.02.006. 26899196

[29] Albinsson S, Suarez Y, Skoura A, Offermanns S, Miano JM, Sessa WC. MicroRNAs are necessary for vascular smooth muscle growth, differentiation, and function. Arterioscler Thromb Vasc Biol. 2010;30(6):1118–26. 10.1161/atvbaha.109.200873. PMC2880481 20378849

[30] Zhou L, Xing C, Zhou D, Yang R, Cai M. Downregulation of lncRNA FGF12-AS2 suppresses the tumorigenesis of NSCLC via sponging miR-188-3p. Open Med (Wars). 2020;15(1):986–96. 10.1515/med-2020-0219. PMC7724005 33344773

[31] Dai B, Jiang J. Increased miR-188-3p in Ovarian Granulosa Cells of Patients with Polycystic Ovary Syndrome. Comput Math Methods Med. 2021;2021:5587412. 10.1155/2021/5587412. PMC8062204 33953792

[32] Li Q, Wang Z, Xing H, Wang Y, Guo Y. Exosomes derived from miR-188-3p-modified adipose-derived mesenchymal stem cells protect Parkinson’s disease. Mol Ther Nucleic Acids. 2021;23:1334–44. 10.1016/j.omtn.2021.01.022. PMC7920810 33717653

[33] Zhang XF, Yang Y, Yang XY, Tong Q. MiR-188-3p upregulation results in the inhibition of macrophage proinflammatory activities and atherosclerosis in ApoE-deficient mice. Thromb Res. 2018;171:55–61. 10.1016/j.thromres.2018.09.043. 30253270

[34] Mi S, Wang P, Lin L. miR-188-3p inhibits vascular smooth muscle cell proliferation and migration by targeting fibroblast growth factor 1 (FGF1). Med Sci Monit. 2020;26:e924394. 10.12659/msm.924394. PMC7547530 33020467

[35] Liao H, Zhang L, Lu S, Li W, Dong W. KIFC3 promotes proliferation, migration, and invasion in colorectal cancer via PI3K/AKT/mTOR signaling pathway. Front Genet. 2022;13:848926. 10.3389/fgene.2022.848926. PMC9257096 35812733

[36] Tian B, Zhao Y, Liang T, Ye X, Li Z, Yan D, et al. Curcumin inhibits urothelial tumor development by suppressing IGF2 and IGF2-mediated PI3K/AKT/mTOR signaling pathway. J Drug Target. 2017;25(7):626–36. 10.1080/1061186x.2017.1306535. 28286973

[37] Wathes DC, Cheng Z, Fenwick MA, Fitzpatrick R, Patton J. Influence of energy balance on the somatotrophic axis and matrix metalloproteinase expression in the endometrium of the postpartum dairy cow. Reproduction. 2011;141(2):269–81. 10.1530/rep-10-0177. PMC3021913 21123519

[38] Nagase H, Visse R, Murphy G. Structure and function of matrix metalloproteinases and TIMPs. Cardiovasc Res. 2006;69(3):562–73. 10.1016/j.cardiores.2005.12.002. 16405877

[39] Wu X, Zheng X, Cheng J, Zhang K, Ma C. LncRNA TUG1 regulates proliferation and apoptosis by regulating miR-148b/IGF2 axis in ox-LDL-stimulated VSMC and HUVEC. Life Sci. 2020;243:117287. 10.1016/j.lfs.2020.117287. 31926240

[40] Wang YC, Hu YW, Sha YH, Gao JJ, Ma X, Li SF, et al. Ox-LDL upregulates IL-6 expression by enhancing NF-κB in an IGF2-dependent manner in THP-1 macrophages. Inflammation. 2015;38(6):2116–23. 10.1007/s10753-015-0194-1. 26063187

